# Supplementary material for: Performance optimization for an optimal operating condition for a shell and heat exchanger using a multi-objective genetic algorithm approach
Source: PLoS One. 2024 Jun 10;19(6):e0304097. doi: 10.1371/journal.pone.0304097 (PMC11164377; doi:10.1371/journal.pone.0304097)
Supplement: Attachment — Submitted filename: Review_Vibhu Sharma.docx [file pone.0304097.s001.docx]

**Review of Performance optimization for an optimal operating condition for a Shell and Heat Exchanger Using a Multi-Objective Genetic Algorithm Approach**

*Report Prepared By: Vibhu Sharma*
*Date: 03/22/2024*

**1. Introduction**

The academic paper under review presents a hybrid optimization method aimed at addressing challenges in thermal engineering. Upon thorough examination, following areas of improvement have been identified to enhance the clarity, coherence, and accessibility of the content.

**2. Analysis of Segments**

The review encompasses an analysis of various segments within the paper, focusing on key aspects such as language usage, structural coherence, and technical clarity.

**Introduction Clarity:** The specific challenge addressed by the hybrid optimization method in thermal engineering can be clearly articulated within the introduction.

**Practical Implications Elaboration:** The discussion section can delve deeper into the practical implications and applications of the findings in thermal engineering and related fields.

**Language Simplification:** While technical terms are used appropriately, the inclusion of complex vocabulary such as "labyrinthine" and "adroitly" may hinder readability. Simplifying language will make the content more accessible to a broader audience.

**Transitional Phrases:** Clear transitions between different topics are necessary to guide the reader through the text seamlessly. Adding transitional phrases or sentences will enhance coherence.
