# Supplementary material for: Performance optimization for an optimal operating condition for a shell and heat exchanger using a multi-objective genetic algorithm approach
Source: PLoS One. 2024 Jun 10;19(6):e0304097. doi: 10.1371/journal.pone.0304097 (PMC11164377; doi:10.1371/journal.pone.0304097)
Supplement: Attachment — Submitted filename: Reviwer Responses.docx [file pone.0304097.s002.docx]

**Title:** Performance optimization for an optimal operating condition for a Shell and Heat Exchanger Using a Multi-Objective Genetic Algorithm Approach

**Reviewer #1:**

Q1. The authors essentially use experiments to set up their predictive models. Therefore, they should improve this part by including details like heat losses analysis from the heat exchanger, that doesn't seem to be properly insulated in Fig. 1, and uncertainty analysis.

**Ans:** Thank you for your Review. In Figure 1, the fabrication process of a portable shell and tube heat exchanger is depicted. During experimentation, meticulous attention was given to insulating the heat exchanger effectively to prevent any heat loss. Following the recommendation, uncertainty analysis has been integrated into the experimental framework.

Q2. The authors introduce the heat exchanger equation (Eq. 1). Are they making references to a tube-in-tube heat exchanger, or did they need to use a F correction factor to account for the shell-and-tube geometry?

**Ans:** Thank you for your Review. In the equation 1, a correction factor has been incorporated to account for specific conditions. However, in the typed version, a general heat exchanger equation was mistakenly used. As per the recommendation, the equation has been adjusted to reflect the appropriate correction factor.

Q3. In subsection 3.2.2, the obtained R2 generally look quite weak for an accurate prediction. Why didn't the authors consider multiple non-linear regression analysis to improve R2? Besides, why the regression looks so weak for the variable TR when compared to the other variables (E and U) shown in subsections 3.2.1 and 3.2.3?

**Ans:** Thank you for your review. Multiple non-linear regression might not have been considered due to its complexity and potential interpretability issues. The weaker performance of the TR variable compared to E and U variables could be attributed to its lesser significance, potential measurement errors, or inappropriate model specifications. Potential solutions include data preprocessing, exploring different transformations for TR, and considering alternative modeling techniques like machine learning.

Q4. The authors claim that they use 16 samples for the regression analysis, including the neural network generation (see subsection 3.3 and Fig. 6). Aren't these too few? This would mean that they just use about 3-4 samples for validation and testing

**Ans:** Thank you for your review. The utilization of a mere 16 samples, inclusive of the Taguchi L16 design featuring 4 levels and 3 factors, may raise concerns regarding the adequacy of the dataset for validation and testing. While the limited sample size may appear restrictive, the Taguchi design allows for a comprehensive exploration of experimental conditions within these constraints, facilitating robust analysis despite its compact nature.

Q5. In Fig. 7 and similar, please use different labels for the figures, since target and output are too generic. Generally speaking, graphics must be improved in this paper

**Ans:** Thank you for your review. In Figure 7 is generated by software (MATLAB).

Q6. The authors generally compare experiments, response surface method, and neural network (see for instance Table 6). Since this might be a novelty here, it is suggested to show a dedicated section that compare pro/cons of each approach, highlighting computational costs and effort for each of the methods. Of course, with references to experiments one might underline the fact that these are by definition costly and long to perform. Similar papers have been recently published about optimization analysis under different regression approaches ([doi.org/10.3390/en15197352](http://doi.org/10.3390/en15197352)) so it is suggested to compare the present outcomes with the ones already available in literature

**Ans:** Thank you for your review, as per your suggestions Manuscript is revised.

Q7. When presenting Fig. 13, the authors should avoid negative variables even if they minimize the negative of a variable. Besides, even if it is not that easy in a 3D space, they should try to show dominated points

**Ans:** Thank you for your review. As per your suggestions, Manuscript is revised.

Q8. Genetic algorithm is generally helpful if one wants to reduce the number of computations to achieve optimum. Therefore, could the authors provide more information about computational time and costs to underline the reason why the genetic algorithm is here necessary? Or did they just assume that since the experimental campaign is generally long then the overall optimization procedure is time costly and using the genetic algorithm might be of help?

**Ans:** Thank you for your review. The Manuscript focuses on determining the optimal input parameters to achieve the best output results.

**Reviewer #2**

Q1. In the introduction, please clarify the specific challenge addressed in thermal engineering. Elaborate on practical implications and applications in the discussion section.

**Ans:** Thank you for your review. As per your suggestions, the Manuscript is revised.

Q2. Simplify language to improve readability. Add transitional phrases for coherence.

**Ans:** Thank you sir for your review. As per your suggestions, the Manuscript is revised thoroughly.
